# Human papillomavirus as a driver of head and neck cancers

**DOI:** 10.1038/s41416-019-0602-7

**Published:** 2019-11-11

**Authors:** Maria Elisa Sabatini, Susanna Chiocca

**Affiliations:** Department of Experimental Oncology, IEO, European Institute of Oncology IRCCS, IFOM-IEO Campus, Via Adamello 16, 20139 Milan, Italy

**Keywords:** Head and neck cancer, Oncology

## Abstract

The human papillomavirus (HPV) family includes more than 170 different types of virus that infect stratified epithelium. High-risk HPV is well established as the primary cause of cervical cancer, but in recent years, a clear role for this virus in other malignancies is also emerging. Indeed, HPV plays a pathogenic role in a subset of head and neck cancers—mostly cancers of the oropharynx—with distinct epidemiological, clinical and molecular characteristics compared with head and neck cancers not caused by HPV. This review summarises our current understanding of HPV in these cancers, specifically detailing HPV infection in head and neck cancers within different racial/ethnic subpopulations, and the differences in various aspects of these diseases between women and men. Finally, we provide an outlook for this disease, in terms of clinical management, and consider the issues of ‘diagnostic biomarkers’ and targeted therapies.

## Background

**Fig. 1:**
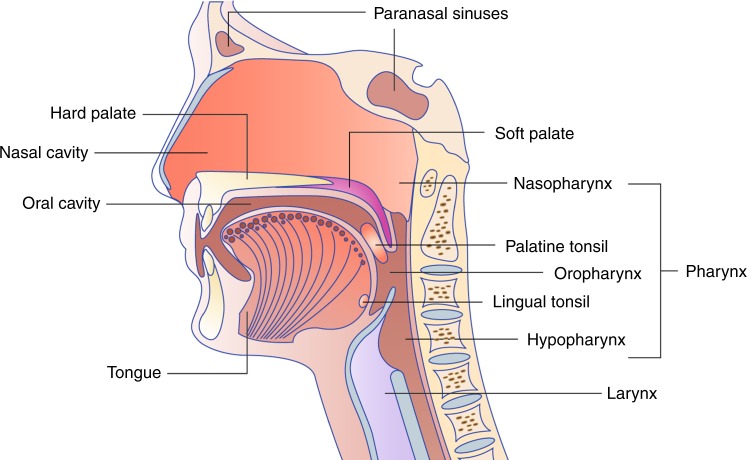
Schematic representation of the head and neck region relevant for head and neck carcinomas.

Head and neck cancer, or head and neck squamous cell carcinoma (HNSCC), comprises a group of malignancies that affect mucosal linings at different anatomic sites of the upper aerodigestive tract, including the nasopharynx, paranasal sinuses, oral cavity, oropharynx, hypopharynx and larynx (Fig. 1). HNSCC accounts for more than 650,000 new cases of cancer annually and more than 350,000 deaths. The classical major risk factors are tobacco and alcohol, and in the past few decades, human papillomavirus (HPV) has emerged as a novel risk factor for these cancers, especially for oropharyngeal squamous cell carcinoma (OPSCC), defining a new subtype of tumour that is distinct from HPV-negative ones. The incidence of HNSCC varies depending on the anatomical region and geographical location. For example, oral cavity and laryngeal cancers are the most common HNSCC globally, and the incidence and mortality rates are higher in Europe compared with the USA (United States of America).^1^ Men are significantly more likely to develop HNSCC than women with an incidence ratio ranging from 2:1 to 4:1.^2^ The average age of diagnosis is 50–70 years (reviewed in^3^). Globally, the incidence of HNSCC has increased by 36.5% over the past decade.^1,4^ A lower socioeconomic status and poor oral hygiene are the characteristics of HPV-negative HNSCC patients. HPV-negative HNSCC also features genomic complexity and very frequent alterations in the tumour suppressor *TP53* and in cell-cycle regulators. These patients associate with unfavourable prognosis and are currently treated with standard care approaches, including cisplatin and radiation. On the contrary, HPV-positive oropharyngeal carcinoma (HNSCC/OPSCC) is generally highly susceptible to radiation and anticancer drugs and has a better prognosis.

A quarter of incident cases of HNSCC comprise OPSCC (reviewed in^5,6^), with HPV being an important implicated risk factor for its development. HPV infection is also associated with a small number of other HNSCC subsites. Data in this regard are quite discrepant, mostly because of insufficient details on anatomical tumour localisation and different HPV detection methods (discussed in the section below). A recent meta-analysis highlighted how the average incidence rates of HPV-positive patients with tumours of other head and neck regions excluding OPSCCs, such as larynx and oral cavity, are generally less than those for oropharyngeal tumours.^7^ The contribution of HPV to HNSCC is common within the tonsillar crypt in the oropharynx. Tonsillar crypt cells, similar to cervical squamocolumnar junction cells,^8^ are organised in a discontinuous single-layered epithelium that is more susceptible to carcinogenic transformation than cells within the oral cavity subsite. Indeed, although HPV infections are more common in the oral cavity than they are in tonsillar crypts, HPV-driven oral cavity squamous cell carcinoma (OCSCC) accounts for only 3.9% of cases, in contrast with the 47% of cases of OPSCC that are attributed to HPV (reviewed in^9^). Other viruses such as Epstein–Barr virus (EBV) and polyomaviruses also have a pathogenic role in HNSCC, sometimes by co-infecting with HPV, but in this review we will focus on our current understanding of HPV in these cancers, since they are currently on the rise and projected to keep increasing. We discuss differences in various aspects of HPV-positive HNSCC between women and men, and within different racial/ethnic subpopulations, before outlining clinical management and considering the issues of biomarkers and targeted therapies.

## The true impact of HPV on head and neck cancer

### HPV infection and carcinogenesis

The oncogenic role of HPV was first elucidated 40 years ago when zur Hausen^10^ discovered a link between the virus and the onset of cervical cancer. Later studies confirmed the direct role of several mucosal HPV types in the development of cervical cancer and other epithelial tumours.^11–14^ HPV infects the stratified squamous epithelia, both cutaneous and mucosal including skin of hands and feet, as well as the anogenital tract, mouth, throat and respiratory tract. Only 3–5% of HPV infection in the cervix gives rise to transforming infection, probably depending on the cell type of origin. The single-layered epithelial cells at the cervical squamocolumnar junctions between the columnar epithelium of the endocervix and the squamous epithelium of the ectocervix are the most susceptible cells for transforming infections (reviewed in^15^). Similarly, the single-layered tonsillar crypts constitute the epithelium with the highest susceptibility to cellular transformation in the head and neck region.^9,16^ These highly invaginated crypts are natural hosts for bacterial infections and foreign materials, driving the expression of programmed cell death-1 ligand-1 (PD-L1).^17^ Since PD-L1 is responsible for immune evasion by binding programmed death-1 (PD-1) receptors expressed by the cells of the immune system,^18^ PD-L1 overexpression in tonsils favours persistent HPV infection allowing tumorigenesis. Furthermore, in tonsillar crypts, the formation of a biofilm composed of bacterial microcolonies encased in a glycocalyx matrix contributes to the ability of HPV to escape the immune system.^19^ On the contrary, the stratified epithelium of the oral cavity, which is easily infected with HPV, is less susceptible to the carcinogenic transformation that leads to the onset of OCSCC.^9^ The HPV family consists of circular, double-stranded DNA viruses of 8000 base pairs encoding proteins involved in viral replication (E1 and E2/E4) and assembly (L1 and L2), as well as accessory proteins (E5, E6 and E7). High-risk HPV types, including HPV16, HPV18, HPV31, HPV33, HPV35, HPV39, HPV45, HPV51, HPV52, HPV56, HPV58, HPV59 and HPV68, can induce carcinogenic transformation of the infected mucosal epithelium (reviewed in^20^) by escaping cell-cycle checkpoints through E6- and E7-mediated degradation of p53 and Rb proteins, respectively (reviewed in^21,22^). E6/E7 expression is frequently associated with integration of the viral genome into DNA regions of genomic instability, followed by disruption of the E2 coding region and dysregulation of E6/E7 themselves. This enables HPV to establish persistent infections and continue to replicate, as the infected epithelial cells are terminally differentiated, but, although viral proteins are synthesised, no viral particles are produced by the virus. This nonproductive infection by HPV is key for the induction of tumorigenesis (reviewed in^23^). Low-risk HPV types induce mucosal infections that are commonly cleared by the host immune system. In the event that they evade innate immunity, low-risk HPVs can cause benign lesions, such as genital warts (HPV1, HPV6 and HPV11), and in sporadic cases, recurrent respiratory papillomatosis (HPV11) with an associated cancer risk (reviewed in^24^). The reduced carcinogenicity of low-risk HPVs is generally due to the low affinity of E6 and E7 towards p53 and Rb, as they are committed to other functions, such as viral episomal maintenance^25^ and induction of apoptosis.^26^ The epithelium of head and neck can also be infected with other tumorigenic viruses such as EBV, BK virus (BKV), John Cunningham virus (JCV) and simian vacuolating virus 40 (SV40), as depicted in the Box 1. In the era of next-generation sequencing (NGS), different types of HPV lineage (1–10% genetic differences) and sub-lineages (0.5–1% genetic differences) have been identified to provide new insights into genetic variants related to carcinogenesis. Following the most recent nomenclature, we can discriminate 16 sub-lineages: A1–A3 (historically classified as European), A4 (Asian), B1–B4 (African-1), C1–C4 (African-2), D1 (North American), D2 and D3 (Asian-American) and D4 (reviewed in^27^). A recent review^28^ summarises lineage-specific and non-lineage-specific HPV16 variants that are associated with HNSCC prevalently detected within E6 and L1 genes. NGS also facilitated the identification of variant lineages of HPV16 that co-infect cervices.^20^ Genetic variants within *E6* regulatory regions or coding sequence may affect gene transcription or induce amino acid exchanges, thus altering the transforming function of this oncoprotein.^28^ For instance, E6 amino acid variations may modify the affinity to the target protein p53, increasing or reducing its tumour-suppressor function. A well-studied example is the T350G *E6* variant, a non-lineage-specific nucleotide change common in tonsillar and cervical cancers. This variant leads to an amino acid change at residue 83 from a leucine to a valine (denoted L83V) and may increase the pathogenicity of HPV16 by enhancing persistency of infection in cervical cancer,^29,30^ although the results are still controversial.^31–33^ In HNSCC, T350G is the most prevalent E6 genomic variant and has a higher viral load compared with the prototype.^34,35^

In contrast, the *E7* genetic sequence is widely conserved in cervical cancer, as demonstrated in a cohort of 5570 HPV16-infected cases.^36^ A reduced capacity to trigger the immune response increases the pathogenic properties of the virus, as described when the *L1* gene harbours genetic variants (reviewed in^37,38^). Although mutations in *L1* gene may reduce viral fitness by impairing capsid assembly, they may contribute to virus persistence by evading the host immune response.^38^ Analysis of 250 prospectively collected cervical cancer tissue biopsies from Indian patients identified 16 major variants (V1–V16) in HPV16 full-length *L1* gene.^39^ This Indian study highlighted major *L1* gene variations coding for amino acid substitutions, such as T379P and L500F, which may play important roles in the immunogenicity against HPV. The T379P mutation affects the binding affinity of the immunogenic peptide (epitope), reducing the host immunogenic response, while the L500F variation induces a stronger immune response than the prototype gene. Similarly, genomic variations within the *L2* coding sequence interfere with antigenic capacity.^40^ Variability of the *L1* or *L2* genes could affect the efficiency of infection and interfere with immunogenicity, with strong implications for the potential development of therapeutic and preventive vaccines and diagnostic tests. A recent study evaluated the association between HPV16 lineages and the risk of cervical precancer/cancer occurrence in 3200 women from a USA cohort.^41^ HPV16 variant lineage association with cervical precancer/cancer lesions (CIN2 and CIN3 separately) compared with the risk calculated for the most common sub-lineage A1/A2 was assessed. Within the ‘European’ variant lineages, the A4 sub-lineage was linked to an increased risk of cancer onset compared with the A1/A2 clade, while among the ‘African’ sub-lineages, the B variant was associated with a reduced risk compared with the A1/A2 variants. Due to the small number of samples, the D lineages were grouped together and also have a higher risk compared with the A1/A2 sub-lineages. This increased precancer/cancer risk results from mutations in the *E6E7 p97* promoter or from a sense gene mutation in the *E1*, *E2* or *L2* sequences, resulting in replication advantage for the virus. Race and ethnicity determine the risk for the development of precancer and cancer in the presence of specific HPV16 variant lineages. Indeed, as reported by Xi et al.,^42^ with an HPV16 European variant infection, white women were more likely to develop CIN3 compared with African–American women. The authors attributed the race/ethnicity-related risk to a diversity in the host immune response due to a co-evolution of the virus with the ‘American’ women and an increased capacity to escape from the immune system.

Box 1: Other viruses related to HNSCC oncogenesis HNSCC can develop in the nasopharynx, a subsite that can also be infected with Epstein–Barr virus (EBV). Natural hosts of EBV are B lymphocytes, epithelial cells and, rarely, smooth muscles of immunodeficient individuals.^112^ Persistent infection can trigger the onset of severe disorders such as Burkitt’s lymphoma,^113^ an uncommon smooth muscle neoplasm,^112^ gastric cancer, squamous cell carcinoma and nasopharyngeal cancer (NPC) (reviewed in^114^). However, in contrast with the western predominance of HPV-related HNSCC, NPC is endemic in a few areas including Southern China, Southeast Asia, North Africa and the Arctic. Co-infection of EBV and HPV in NPC patients has been detected at frequencies ranging from 10 to 47% in endemic NPC cohorts, whereas several studies have suggested that high-risk HPV and EBV are mutually exclusive in NPC cases from non-endemic areas (reviewed in^115^). Synergistic effects of the two viruses in NPC cannot be supported as the rate of co-infection is low and controversial among different studies. A similar debate surrounds the role of EBV in HPV-positive OSCC (oral SCC, which includes the oropharynx and oral cavity). Polz-Gruszka et al.^116^ reported a very low rate of co-infection in their cohort of patients, whereas Jiang et al.^117^ suggested a carcinogenic role for HPV–EBV co-infection in lymphoid-rich oropharynx sites. In a recent study,^118^ co-infection of HPV–EBV in OSCC was observed in 34% of cases, especially in smokers, and correlates with N1 and N2 stages (N, node; defines whether the tumour has spread to the lymph nodes and how many lymph nodes are involved), as well as poorly differentiated tumours (G3, where G defines the grade of tumour). On the other hand, EBV single infection is possibly associated with OSCC, as demonstrated in a recent meta-analysis.^119^ A few studies have investigated the role of polyomaviruses in HNSCC oncogenesis, both independently or as a co-factor for HPV.^120–122^ Primary infection mostly occurs during childhood, but the virus remains quiescent in kidney or lymphoid tissues as long as the immune system is not compromised. Polyomaviruses include BKV, which induces haemorrhagic cystitis and nephropathy, JCV, which can cause progressive multifocal leukoencephalopathy in immunocompromised patients, and SV40, which is oncogenic for the onset of primary brain and bone cancers, malignant mesothelioma and lymphomas in laboratory animals.^120,123^ JCV and SV40 DNA have been detected in oropharynx, nasopharynx and hypopharynx, larynx, lip, tongue and oral cavity cancers.^120,122,123^ JCV DNA was detected in 37% of HNSCC samples at a high copy number;^120^ however, viral load was unrelated to tumour grade. By contrast, SV40 DNA was present in only 13.4% of samples with a low copy number, whereas BKV-positive patients account for 1.2%. Polyomaviruses have been found in concert with HPV in HNSCC, although co-infection did not affect disease-specific overall survival. In contrast with the results described by Poluschkin et al.,^120^ Drop et al.^118^ estimate an HPV–BKV co-infection in 22.0% of patients, while 20.7% of patients were simultaneously infected with HPV, BKV and EBV. BKV–EBV co-infection and HPV–BKV–EBV co-infection highly increases the frequency of poorly differentiated tumours (G3) compared with patients with only EBV infection.

### Detection of HPV

The use of different viral detection methods between different studies might overestimate or underestimate the number of patients affected with virus-related HNSCC. Viral positivity is often determined by viral DNA detection through PCR techniques, but this approach might indicate a transient infection that is unrelated to carcinogenesis.^16^ Several markers can be used for HPV detection besides PCR, including E6/E7 HPV mRNA RT-PCR,^43^ HPV DNA in situ hybridisation and p16^INK4a^ (referred to herein as p16) in situ hybridisation, as reviewed by Taberna et al.^44^ During tumorigenesis, the HPV E6 and E7 oncoproteins decrease the levels of p53 and functional Rb by post-translational regulation, leading to aberrant overexpression of the cell-cycle protein p16,^45^ which can be detected also by immunohistochemistry (IHC),^46^ rationalising its use as a surrogate marker for the presence of high-risk HPV. Indeed, p16 IHC has become the recommended stand-alone prognostic test for patients with OPSCC, as underlined in the new 8th edition American Joint Commission on Cancer (AJCC) staging guidelines where p16 IHC is required to stage OPSCC patients.^47^ This technique is continuously under revision to optimise one specific antibody and a common platform for routine clinical use.^48^ Nevertheless, it does not evaluate whether the virus is transcriptionally active, which can be evaluated by HPV E6*I mRNA expression, a marker of transcriptional activity of HPV oncogenes.^49^ Nevertheless, p16 detection is a good indicator for tumour status among OPSCC patients but not for non-OPSCC patients.^50^ As a tumour suppressor, p16 expression is frequently lost in most cancers, including HPV-negative HNSCC, due to gene mutations, deletions or promoter methylation.^51^ p16 silencing has been previously associated with tobacco exposure,^52^ one of the pivotal risk factors for HPV-unrelated HNSCC. Indeed, p16 loss is frequent in patients who smoke, indicating that this marker should be used carefully in the co-presence of risk factors. Similarly, alcohol consumption is correlated to p16 loss, as demonstrated in a Japanese study where 106 out of 137 OPSCC patients who drank were p16-negative, and among non-drinkers only 16 out of 36 were p16-negative.^53^ Thus, in non-OPSCC patients, mostly HPV negative, other risk factors can affect p16 status. Remarkable evidence has been reported in a 2017 study^54^ of Indian patients with HNSCC, in which there was an impressive discrepancy between HPV positivity and p16 detection: more than half of HPV DNA/RNA-positive cases were found to be p16-negative. Furthermore, a recent study indicates that the role of p16 in non-OPSCC patients might not be related to HPV positivity, as it is in OPSCC patients,^55^ in contrast with the results of Bryant et al.,^56^ which suggest a similar prognostic role for p16 for both types of cancer. Although HPV has been extensively considered a factor of good prognosis for HNSCC, the optimal way to predict the clinical outcome is to evaluate more than one biomarker. Smith et al.^57^ suggested a joint group of biomarkers, including HPV, p16 and p53, to predict the survival of HNSCC patients. Taken together, we can conclude that studies that took into account just one marker for HPV positivity should be critically re-analysed, and that the goal of finding a true surrogate biomarker for HPV positivity is still a challenge.

### Clinical and epidemiological characteristics of HPV-driven HNSCC

Compared with HPV-negative HNSCC, HPV-positive HNSCC is characterised by a simpler genomic mutational load, since p53 and Rb are silenced by the viral oncoproteins E6 and E7, but have frequent activating mutations of genes involved in phosphoinositide 3-kinase (PI3K) pathway (reviewed in^21,22^). HPV-driven HNSCC mainly comprises cancers arising in the oropharynx (OPSCC) and in the tonsils.^58,59^ The incidence of HPV-associated OPSCC has been increasing sharply, particularly in younger age groups with no or very little tobacco exposure, and mostly in North America and northern Europe (reviewed in^60^), and is currently the most frequent HPV-driven cancer in the USA.^61^ Recent studies identified an increase in the elderly population (>70 years of age) of HPV-positive OPSCC.^62^ Trends in its incidence are variable and dependent on region (reviewed in^63^). In contrast with HPV-negative HNSCC, HPV-positive OPSCC has a favourable prognosis, with 5-year survival rates of 75–80%, as these tumours respond better to chemo-/radiotherapy. This favourable prognosis has led to an update to the 8th edition of the AJCC staging system where HPV-positive and -negative OPSCC are separated.^64^

HPV is sexually acquired, and early sexual debut as well as a high number of sexual partners, including oral sex partners, and previous genital warts, provide an increased risk for HPV-positive OPSCC (reviewed in^65^). In addition, there is a higher prevalence of HPV-positive OPSCC in men compared with women, and white populations compared with Asians and black populations.

## Stratification of HPV-related HNSCC by sex

### Differences in prevalence and prognosis of HPV-positive tumours between men and women

A retrospective study of 240 patients with OPSCC diagnosed between 1995 and 2012 at two comprehensive cancer centres in the USA showed that the prevalence of OPSCC is more than twofold higher in men (161 out of 240) than in women (79 out of 240) (Table 1). However, HPV is the major driver of this type of cancer in both sexes. Indeed, 62% of male OPSCC patients and 56% of female OPSCC patients have HPV-positive tumours.^50^ It is worth noting that the proportion of HPV-driven cases of OPSCC increased significantly in the considered time frame not only among men (from 36 to 72%), but also among women (from 29 to 77%)^50^ in the USA. Furthermore, an international study including patients from 29 countries estimated a higher percentage of OPSCC cases that are HPV-associated in women than in men in almost all of the European sub-regions.^16^ In conclusion, although the prevalence of both HPV-positive and HPV-negative OPSCC is higher in men than in women (Table 1), the percentage of HPV-attributed OPSCC is increasing in both sexes, especially in women. By contrast, there is a low percentage of HPV-driven non-OPSCC (10% for p16 positivity), the most including OCSCC, in both women and men,^66^ as mentioned above.

**Table 1:** Differences in diagnosis and prognosis of HPV-positive and HPV-negative oropharyngeal squamous cell carcinoma (OPSCC) and oral cavity squamous cell carcinoma (OCSCC) sorted by sex and based on the most recent USA population studies^50,66,67^

*OPSCC* oropharyngeal squamous cell carcinoma, *HPV* human papillomavirus

A more recent population study on data retrieved from the National Cancer Database (NCDB) from 2010 to 2014, representing over 70% of patients in the USA, shows a higher HPV positivity in men than in women for both OPSCC (66% vs. 50%) and OCSCC (16% vs. 11%).^67^ In a cohort of 21,627 OPSCC patients, the prevalence of HPV-driven tumours is 6.3-fold higher in men than in women, whereas considering the 9080 OCSCC patients the ratio is reduced to a 2.6-fold change (Table 1). A higher prevalence in men has also been observed for HPV-negative OPSCC or OCSCC patients, albeit to a lesser extent (Table 1).^67^

Within all four categories of patients (HPV-positive OPSCC and OCSCC, and HPV-negative OPSCC and OCSCC), women are older at the age of diagnosis, but have cancers that are diagnosed at earlier tumour (T) and node (N) stages, compared with men.^67^ In HPV-negative OPSCC cases, Kaplan–Meier analysis shows a significantly higher overall survival (OS) in men than in women (Table 1), diverging from a previous report,^68^ although in that study patients were not stratified by HPV. According to the Kaplan–Meier analysis, the hazard ratio (HR, 95% CI [confidence interval])—that is, the relative risk of death of a treated group compared with a control group—in HPV-negative OPSCC is significantly higher in women than in men, while no significant differences have been detected in HPV-positive OPSCC (Table 1). By contrast, among patients affected with OCSCC, women have a more favourable OS and lower HR (95% CI) than men, independently of HPV,^67^ in accordance with a previous study (Table 1).^68^ Thus, in OPSCC, the combination of sex and HPV-positivity status can be considered a prognostic factor, whereas sex is a prognostic factor in OCSCC even without accounting for HPV status. These findings might be misrepresented, however, as the study just takes into account the OS of patients without specification of the causes of death, which could be indirectly correlated with cancer. Secondly, data on general cancer risk factors that, at least partially, are gender-associated, have not been considered.

### Potential reasons for gender differences in the prevalence of HPV-positive tumours

Saunders et al.^69^ recently hypothesised that the difference in HPV-positive HNSCC cases between men and women could be caused by the higher rates of HPV transmission for vaginal–oral rather than penile–oral sex. In accordance, another study found that HPV may be transmitted more often from women to men than from men to women, suggesting that transmission rates may differ by sex, although data from literature are still ambiguous regarding male-to-female and female-to-male transmission rates.^70–73^ Generally, sexual behaviour and gender differences in lifestyle, including tobacco smoking and alcohol drinking, cannot fully explain the reported differences in the prevalence of HPV-related HNSCC between sexes.^74^

Hormonal factors are emerging as key players that confer protection against cancer in females. The presence of oestrogen-related and progesterone-related factors, both endogenously and exogenously derived, is inversely correlated with the risk of HNSCC.^75^ Exogenously derived hormones inversely correlated to HNSCC risk, such as oral contraceptives (OC) use and hormone replacement therapy (HRT), defined as hormone therapy for menopausal symptoms. On the contrary, a fluctuation of endogenous hormones is correlated with women’s distinctive life stages. Within the cohort of case studies, data showed that women who had their first pregnancy before 35 years of age have a lower risk of HNSCC in comparison with women who have either never been pregnant or were pregnant after 35 years of age. Similarly, a higher risk of HNSCC is associated with menopause onset before 52 years of age.^75^ Furthermore, oestrogen-related and progesterone-related factors interact with common risk factors, such as smoking and alcohol, that are known to biologically affect female hormone pathways. Indeed, tobacco smoking increases oestrogen catabolism,^76^ while liver metabolism, affected by alcohol drinking, is involved in oestrogen homoeostasis.^77^ The higher oestrogen levels present in alcohol drinkers can act synergistically with the presence of endogenous/exogenous hormones to decrease the HNSCC risk in women.^75^

It is most likely, however, that there are intrinsic biochemical and molecular differences between female and male cells that have an impact on tumorigenesis, independently of sex hormones. For example, differences in the global demethylation pattern of induced pluripotent stem cells have been observed between males and females,^78^ and it is well established that HPV modifies the epigenetic landscape of host cells by upregulating DNA methyltransferase 1 (DNMT1).^79,80^ Furthermore, Hurst et al.^81^ identified a sex-associated mutation within the *KDM6A* gene, which encodes lysine demethylase 6A, in bladder tumours, with a higher mutation frequency in females than in males, painting a gender-specific epigenetic landscape. Thus, given the molecular differences between males and females,^78,81^ we could speculate that the epigenetic landscape might be differently affected by the virus depending on sex. Finally, evidence supports the presence of a sex-driven dimorphism in the immune system in favour of women, thus potentially allowing a rapid clearance of pathogens such as HPV (reviewed in^82^). A systematic review highlighted that antibodies intrinsically produced during HPV16 infection provide modest protection in females against subsequent HPV-mediated genital infections.^83^ No evidence of natural acquired immunity has been reported in men, nor in men who have sex with other men.^84^

## HPV-driven HNSCC in different racial/ethnic subpopulations

HPV-related HNSCC cannot only be stratified by sex but also by race/ethnicity. To date, the majority of studies have investigated the epidemiology of HNSCC primarily in white males, the most affected subgroup within the patient population, although the overall proportion of HPV-positive HNSCC in the past few decades has increased both among men and women, as well as in whites and non-whites. In the case of OPSCC, white patients have the highest prevalence of high-risk HPV-positive cancer onset, followed by blacks and then Asians (as shown in Table 2). In accordance, although HNSCC has the highest incidence in the Indian subcontinent, the contribution of HPV infection is very limited for this population.^54^

**Table 2:** Differences in diagnosis and prognosis of HPV-positive and HPV-negative oropharyngeal squamous cell carcinoma (OPSCC) sorted by race, including Blacks, Whites and Asians

	Blacks	Whites	Asians
HPV-prevalent strain	HPV18	HPV16	HPV16
HPV–p16 correlation	No	Yes	No
HPV + OPSCC onset	+	++	+
HPV + OPSCC survival rate	+	++	+
Factor of bad prognosis	p16 negativity	hrHPV negativity	p16 negativity

*OPSCC* oropharyngeal squamous cell carcinoma, *HPV* human papillomavirus, *hrHPV* high-risk HPC

### Ethnicity, HPV and p16 status

HPV16 is the most predominant genotype for both OPSCC (46.6%) and non-OP HNSCC (13.4%). However, black patients had the highest incidence of HPV18 in OPSCC in comparison with other races^85^ (Table 2), and the reason for this is still under debate. A crucial element that differentiates patients among races, and that is strictly connected with the prognosis of the disease, is the combination of p16 expression and high-risk HPV-positive DNA status. A total of 52.3% of white patients had high-risk HPV positivity in combination with p16 expression, in contrast with a lower percentage of Asian (23%) and black (22.6%) patients (Table 2).^85^ In accordance with these findings, as mentioned previously, Gheit et al.^54^ reported a high discrepancy between p16 positivity and HPV status in the Indian population. Moreover, black patients had a higher proportion of high-risk HPV-positive/p16-negative OPSCC, while Asians accounted for the largest numbers of high-risk HPV-negative/p16-negative OPSCC patients. For white patients, a poor prognosis, including risk of death or recurrence/metastasis, is more likely to be linked with a negative high-risk HPV status, whereas for non-white patients a poor prognosis is associated with negative p16 expression.^85^ The results from this meta-analysis highlighted a lower rate of HPV-driven OPSCC among non-white people, but a more favourable outcome of the disease in white patients than in black or Asian patients (Table 2). Similarly, data extrapolated by the NCDB from a cohort of 22,693 patients from the USA show the highest percentage of HPV-positive OPSCC patients in whites (67.6%), followed by Hispanics (57.1%), Asians (52.9%) and blacks (42.3%).^86^ Data have not been stratified for HPV detection method, and this may explain the discrepancy with the work described previously. Secondly, the high percentage of HPV-driven OPSCC observed in Asian and Hispanic patients probably reflects that, with the exception of black patients, non-white patients living in the USA acquired sexual behaviours similar to white patients. However, another study on OPSCC cases from white, Asian, Hispanic and black patients living in the USA depicted a similar scenario. D’Souza et al.^50^ reported that the majority of OPSCC cases among whites (71%), Asians (86%) and Hispanics (71%) determined by p16 detection were HPV positive, whereas only 40% of black patients with OPSCC were HPV positive.

### Possible reasons for differences in HPV-driven HNSCC in racial/ethnic subpopulations and implications for prognosis

Differences in oral sexual behaviour can partially explain the epidemiological differences in HPV infection across different racial groups.^74^ However, genetic or epigenetic variants amongst different races/ethnicities must be taken into account—for example, black people have a lower proportion of oropharyngeal HPV16 infection, but a higher proportion of HPV18 infection in comparison with the other groups.^85^

The higher susceptibility of black people to HNSCC compared with white people has diminished over the past 20 years, while the lower survival rate of this population has remained, independently of the stage of diagnosis, treatment or sex.^87^ The racial disparity in cancer prognosis is related to genetic and epigenetic diversity, a higher metabolic propensity to obesity and the chronic inflammation status of black people, as well as differences in innate immunity, as reviewed by Ozdemir and Dotto.^88^

A recent study highlighted the differential expression of genes involved in tumorigenesis in European–American versus African–American patients affected with HPV-active and HPV-inactive HNSCC,^89^ considering HPV-active when the HPV DNA is present and transcribes for the oncogenes, and HPV-inactive when HPV DNA is present but is not transcribed, as discussed above. As the authors speculate that HPV-inactive tumours represent an advanced status of HPV-positive HNSCC, different gene expression patterns among black and white people could represent a different progression of the disease among races. It is known that differential gene expression is associated with diversity in cancer aggressiveness with possible clinical implications.^90^ For example, alteration in copy-number amplifications (CNAs), including 3q, 5p and 11q amplification in HNSCC, associated with alterations in expression of genes involved in biologically and therapeutically relevant pathways, results in worse prognosis. Therefore, clustering different races for their gene expression may be relevant to design race-specific therapeutic approaches.

## Outlook

Several biological markers of HNSCC development and progression have been described. These biomarkers are involved in pathways that are differentially affected or modified during the onset of HNSCC, and can be potentially exploited to stratify the patient population in terms of diagnosis and prognosis. The translation of biomarker candidates to the clinic as prognostic/diagnostic factors for patients, however, is still a challenge, as scientific results on this issue are derived mostly from molecular studies on model systems, such as cell lines,^90,91^ very few patient-derived xenograft (PDX)-derived findings^92–94^ or a limited number of patient samples (reviewed in^9^). However, with the increased knowledge on the detailed genomic characterisation of HNSCC, precision medicine can be part of the clinical management of these patients in the near future.

For example, Hussein et al.^95^ validated biomarkers for tongue squamous cell carcinoma (TSCC), a subsite within the oral cavity, by cross-analysing PubMed articles published from 2010 to 2017 in a systematic review. Starting from 1429 research articles, the authors selected 96 studies, and following rigorous inclusion/exclusion criteria, validated ten promising biomarkers for TSCC. Sample sources were both body fluids and tissues derived from the mobile part of the tongue, and the clinical relevance of these indicators has been described in at least two studies. The ‘final ten’ include *TP53* and *NOTCH1* as genetic markers, *MALAT1* as a long noncoding RNA marker, hypoxia-inducible factor (HIF)-1α, SOX2, E-cadherin and vimentin as IHC markers, and interleukin (IL)-6, IL-8 and prolactin as body fluid markers. As these markers are indicators of poor prognosis in TSCC, they could be used to predict a specific progression of malignancy and suggest a possible requirement for treatment intensification for these patients. Along the same lines, an miRNA-based molecular signature to stratify HPV-driven HNSCC patients according to risk of recurrence has recently been identified.^95^ The miRNA expression profiles of samples from 162 patients treated with chemoradiation and surgery in eight different hospitals were analysed, and the transcription levels of five miRNAs (hsa-let-7g-3p, hsa-miR-6508-5p, hsa-miR-210-5p, hsa-miR-4306 and hsa-miR-7161-3p), together with TNM T stage, extracapsular extension (ECE) and TNM N stage, identified four different recurrence risk groups: ‘low-risk’, ‘low–intermediate-risk’, ‘high–intermediate-risk’ and ‘high-risk’. As a consequence, the five-miRNA signature opens the possibility for personalised treatments by adjusting the intensity of therapy according to the overall risk for therapy failure.

In contrast with treatment intensification, clinical trials for treatment de-intensification to reduce long-term potential toxicity are currently being evaluated for HPV-positive OPSCC patients, in view of their better prognosis, compared with their HPV-negative counterparts. First, radiation combined with the less-toxic cetuximab instead of cisplatin, although the De-ESCALaTE trial already suggests caution;^96^ second, induction chemotherapy followed by decreased radiation doses and/or volumes for good responders; third, radiation alone instead of chemoradiation; and, finally, transoral surgery followed, or not, by postoperative radiotherapy depending on the presence or not of risk factors (reviewed in ref. ^97^).

By determining an optimal treatment and follow-up for patients of all ages, sex and ethnicity can help maximise the use of healthcare. Very recently a new trend in the age at diagnosis of OPSCC has been reported.^62^ Indeed, as mentioned above, a dramatic increase in the prevalence of HPV among patients ≥ 70 years of age has been observed over the period 2010–2014, together with a decrease in the survival advantage in HPV-positive OPSCC patients compared with non-OP HNSCC patients due to the increased age. Older HPV-positive OPSCC patients have distinct characteristics that influence treatment response compared with younger patients. For example, a higher comorbidity score and treatment-related toxicities compared with younger patients^98,99^ decrease the survival benefit of possible treatment intensification,^100^ as well as increase the risk of non-cancer deaths.^101^ Accordingly, new trends in the treatment of HPV-positive tumours should take into account the increasing proportion of the elderly population and their distinct clinical characteristics and prognosis.

Similarly, a 2018 systematic meta-analysis outlined a difference in the efficacy of immune checkpoint inhibitors between men and women.^102^ Immunotherapy is emerging as a new strategy to target immunogenic malignancies (reviewed in^103^) such as HNSCC, especially in recurrent and metastatic tumours with very poor prognosis (reviewed in^104^). Both HPV-positive and HPV-negative tumours display a high level of immune checkpoint components, such as PD-1 and cytotoxic T-lymphocyte protein 4 (CTLA-4).^105^ A smaller benefit induced by immunotherapeutic treatments has been observed in females in comparison with males for different tumour types, including HNSCC. The HR (95% CI) spans from 1.4- to 1.7-fold higher in women than in men, regardless of HPV positivity (reviewed in^102^). The higher efficacy in males than in females of immune checkpoint inhibitors as cancer treatment reflects sex-based immunological differences. Generally, adult females have a stronger innate and adaptive immune response than males and a higher vaccine efficacy (reviewed in^82^). On the other hand, this strong immunity contributes to an increased susceptibility to autoimmune diseases (reviewed in^106,107^). Conforti et al.^102^ suggested that the higher efficacy of immunotherapy in men derived from a lower immunogenicity of tumours in females that can escape a stronger immune system, thus resulting in less efficacy of immune checkpoint inhibitors in these tumours. Furthermore, the increased susceptibility of women to autoimmune disorders could trigger the onset of immune checkpoint inhibitor-related adverse events, potentially leading to a higher rate of treatment discontinuation.^108^ This topic is still under debate since, more recently, a meta-analysis demonstrated no statistically significant association of patients’ sex with the effectiveness of immunotherapy,^109^ and the authors criticised the analysis tools used by Conforti et al.^102^ Indeed, another report demonstrated that the blockage of B7-H1, a co-signalling molecule that can hinder antitumour immunity, is more efficient in females compared with males.^110^ Future trials should therefore include larger female populations to erroneously avoid extrapolation of the results in females, and secondly, to improve clinical outcomes of immunotherapy in women. A surprising issue that has emerged from a study in California denotes that female patients are undertreated, as they are less likely to receive severe chemotherapy and radiation compared with their male counterparts, resulting in higher death rates^111^ (Table 1). The reason for this outcome is unknown and hypothesised to relate to an unconscious physician bias, as well as a possible diversity in patient treatment goals.^111^

In summary, the HPV status classifies two distinct entities of HNSCC and is the only clinically validated biomarker for survival in these cancers. Although HPV-positive and HPV-negative HNSCC have significantly different disease profiles, their treatment options do not yet mirror these diversities. However, actionable biomarkers, such as epidermal growth factor receptor (EGFR), PI3K pathway components and possible oncogene abnormalities, are currently being studied by many laboratories in different countries to provide mechanistic evidence for future potential targeted therapy, which can benefit subgroups of both HPV-positive and HPV-negative HNSCC patients. Moreover, due to the genetic and epigenetic diversities, as well as differences in sexual behaviours and risk factors, the need to set-up clinical trials for HNSCC chemotherapies stratified for sex and race is emerging.

## Data Availability

Not applicable.
